# Time-Aware and Temperature-Aware Fire Evacuation Path Algorithm in IoT-Enabled Multi-Story Multi-Exit Buildings

**DOI:** 10.3390/s21010111

**Published:** 2020-12-26

**Authors:** Hong-Hsu Yen, Cheng-Han Lin, Hung-Wei Tsao

**Affiliations:** Department of Information Management, Shih Hsin University, Taipei 116, Taiwan; a107221096@mail.shu.edu.tw (C.-H.L.); a107221014@mail.shu.edu.tw (H.-W.T.)

**Keywords:** evacuation path, multi-story multi-exit building, temperature sensors, multi-time-slots planning, optimization

## Abstract

Temperature sensors with a communication capability can help monitor and report temperature values to a control station, which enables dynamic and real-time evacuation paths in fire emergencies. As compared to traditional approaches that identify a one-shot fire evacuation path, in this paper, we develop an intelligent algorithm that can identify time-aware and temperature-aware fire evacuation paths by considering temperature changes at different time slots in multi-story and multi-exit buildings. We first propose a method that can map three-dimensional multi-story multi-exit buildings into a two-dimensional graph. Then, a mathematical optimization model is proposed to capture this time-aware and temperature-aware evacuation path problem in multi-story multi-exit buildings. Six fire evacuation algorithms (BFS, SP, DBFS, TABFS, TASP and TADBFS) are proposed to identify the efficient evacuation path. The first three algorithms that do not address human temperature limit constraints can be used by rescue robots or firemen with fire-proof suits. The last three algorithms that address human temperature limit constraints can be used by evacuees in terms of total time slots and total temperature on the evacuation path. In the computational experiments, the open space building and the Taipei 101 Shopping Mall are all tested to verify the solution quality of these six algorithms. From the computational results, TABFS, TASP and TADBF identify almost the same evacuation path in open space building and the Taipei 101 Shopping Mall. BFS, SP DBFS can locate marginally better results in terms of evacuation time and total temperature on the evacuation path. When considering evacuating a group of evacuees, the computational time of the evacuation algorithm is very important in a time-limited evacuation process. Considering the extreme case of seven fires in eight emergency exits in the Taipei 101 Shopping Mall, the golden window for evacuation is 15 time slots. Only TABFS and TADBFS are applicable to evacuate 1200 people in the Taipei 101 Shopping Mall when one time slot is setting as one minute. The computational results show that the capacity limit for the Taipei 101 Shopping Mall is 800 people in the extreme case of seven fires. In this case, when the number of people in the building is less than 700, TADBFS should be adopted. When the number of people in the building is greater than 700, TABFS can evacuate more people than TADBFS. Besides identifying an efficient evacuation path, another significant contribution of this paper is to identify the best sensor density deployment at large buildings like the Taipei 101 Shopping Mall in considering the fire evacuation.

## 1. Introduction

Fire detection and evacuation are an important issue in today’s multi-floor building construction. In the existing evacuation approach, evacuees in the building are evacuated by following the evacuation sign on the wall to the emergency exits. However, evacuees following the fixed evacuation sign to the nearest emergency may be stuck on the route when the fire spread to the emergency route. This calls for an intelligent evacuation path that can bypass the fire zone to the emergency exit in a timely manner. In a contemporary multi-floor building, temperature sensors and smoke sensors are deployed to detect and issue an alarm when there is a fire or smoke in the building. These sensors are connected by wired or wireless networks to enable the IoT capabilities in monitoring and capturing the fire signal in the buildings. When the fire signal is detected, the most important thing is to identify the evacuation path in the buildings.

The temperature data collected by the temperature sensors deployed in the building can provide useful information for planning the evacuation path. Basically, the temperature sensors will periodically sense, collect and transmit the temperature data back to the control station, also known as the sink node. Instead of a traditional fixed evacuation path, a temperature-aware evacuation path should be designed to provide a safer evacuation route by using the temperature data collected by the sensors. In a fire zone that has a very high-temperature or is full of inflammable materials (e.g., paper, wood, plastic), the fire spreading can be very fast so as to increase the temperature dramatically in a very short period of time. In addition, evacuation in a large multi-floor building should adapt to the temperature changes because of longer evacuation time. Hence, the evacuation path should be re-calculated and rerouted at every time slot to acquire the most recent temperature data.

Evacuation path planning has been studied in recent years; most focus on the evacuation path planning in a two-dimensional graph. However, these algorithms are not applicable to the three-dimensional multi-floor buildings. In addition, most of the previous research calculates the evacuation path based on one-shot temperature data captured by the sensors. This one-shot temperature data calculated evacuation path is not valid in the large multi-floor buildings where the temperature can change dramatically in the fire environments during evacuation.

According to the fire research in [1], fire progression rapidly in minutes. The temperature can reach 500 °F within three to four minutes in the incipient stage of today’s fire environments. The flashover stage that is over 1100 °F can develop under five minutes. It is reported in [1] that the upper limit of human temperature tenability is 212 °F or 100 °C. In addition, the experiments in [2] show that deaths by a thermal injury occurring at a temperature of 350 °F in three minutes. Another result in [3] shows that the critical time for the fire is 5.5 min; after this time, the fire spread quickly to the other parts of the building. This indicates that the people evacuation time window is very limited. In addition, the temperature increasing rate on the evacuation path may be faster than the people evacuation time, so the evacuation path should be adaptable based on current temperature data. In other words, the evacuation path should be adjusted to bypass the fire zone based on temperature data acquired by the sensors at the current time slot. Evacuation at large multi-floor buildings needs more time than the evacuation path algorithm should address the fire spreading on a timely basis. This calls for a new three-dimensional evacuation path planning algorithm that can adapt its path based on most current temperature data from the sensors.

Note that most injuries and deaths are caused by smoke in the fire. Human exposes to toxic gases (e.g., carbon monoxide concentration of 0.1% to 0.8%) within one or two minutes will become incapacitated in movement [4]. In [5], they show that there is a linear relationship between temperature rise and carbon monoxide concentration in the fire. Hence, the higher temperature, the higher the carbon monoxide concentration. In this paper, without loss of generality, the temperature data acquired by the temperature sensor is used as the hazard index of toxic smoke concentration and the heat radiation from the flame.

**Figure 1:**
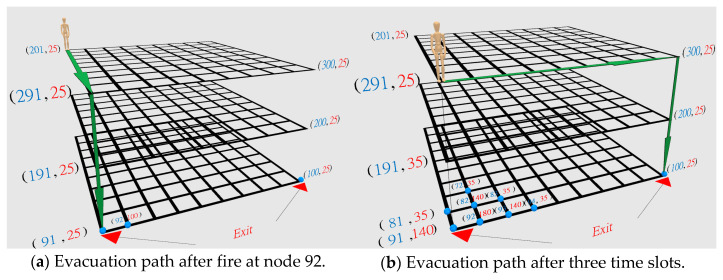
Evacuation path adaptable to temperature changes in a three-story two-exit building.

Figure 1 shows an illustrative example of evacuation path planning in different time slots in a three-floor building where each floor has a grid size of 10 × 10. In this example, 100 temperature sensors are deployed at each floor, and 300 deployed sensors are deployed in the three-floor building. How to determine the grid size (or sensor density) at each floor depends on the tradeoff between evacuation speed and fire spreading speed. In Section 5, we will show that the proposed algorithms can help to determine the best density of deployed temperature sensors in the building.

In Figure 1, the number in blue color indicates the node ID, and the number in red color indicates the temperature at that node. In Figure 1a, the fire is first detected at node 92, and the evacuation path is shown as the green color line from node ID 201→291→91 at the first time slot. In Figure 1b, we assume people can move at most three hops distance in one time slot. Then after three time slots, the user can move from location 201 to location 291. At the same time, we also assume the fire spreads to the neighbor nodes in one hop distance at each time slot. After three time slots, the temperature at the emergency exit at node 91 is 140 °C. In this case, a new evacuation path should be identified because the temperature at location 91 is higher than the upper limit of human temperature tenability, 100 °C. The new evacuation path is shown as the green line in Figure 1b. Then an interesting research question is how to design and plan a minimum total temperature and a minimum evacuation time evacuation path in considering the temperature changes in the different time slots.

In this paper, we first propose the mathematical models to capture this evacuation path planning problem in the multi-story multi-exit buildings and then develop the temperature-aware and time-aware algorithms based on the temperature data periodically captured by the temperature sensors. In the proposed mathematical models and evacuation path algorithms, we generalized the path cost metric to address the time cost and the temperature cost. When using the total time slots as the cost metric, the proposed evacuation path algorithm identifies the path that can evacuate people as fast as possible and in-the-meantime to bypass the fire zone. When using the total temperature on the evacuation path as the cost metric, the proposed evacuation path algorithms identify the comfortable evacuation path with the minimum total temperature on the evacuation path.

The contribution of this paper is the proposed multi-time-slot-aware evacuation algorithm that can help the evacuees to evacuate in the most efficient way. To distribute the calculated evacuation path to the evacuee on a timely basis, another three components should be constructed in the system. The first is the temperature sensors that will report the sensing temperature data back to the control center. The second is the control center that calculates the evacuation path by using the proposed algorithms for each user and distribute the evacuation path to the user’s app. The third is the user’s app that shows the evacuation path from the control center and reports his/her location to the nearby Wi-Fi APs.

We summarize the contributions of this paper.

- Novel time and temperature aware evacuation path (TTAEP) model: We propose a rigorous mathematical model, TTAEP, to capture the evacuation path planning problem in the multi-story multi-exit building with addressing to the temperature changes in different time slots.
- Novel time aware and temperature aware evacuation path planning algorithm: Six evacuation algorithms are devised to tackle the TTAEP problem where the objective is to identify the evacuation path with minimum evacuation time and minimum total temperature on the path. The basic idea of the proposed algorithms is to identify the multi-staged best paths to adapt to the temperature changes at each stage. By using the most recent sensed temperature data return from the sensors, the proposed algorithms identify the multi-staged evacuation path.
- Helps to determine the density of deploying temperature sensors in the building: From the computational experiments in the Taipei 101 Shopping Mall, we observe that the density of deployed temperature sensors in the building plays a non-negligible role in determining the efficiency of the evacuation paths and the best sensor density should consider the tradeoff between evacuation speed and fire spreading speed. The computational experiments of the proposed algorithms can help to determine the best density of deployed temperature sensors in the building.
- Helps to determine the maximum people capacity in the building: From the computational experiments in the Taipei 101 Shopping Mall, we observe that at most 800 people can be successfully evacuated within 15 time slots in the extreme case of seven fires. The computational experiments of the proposed algorithms can help to determine the maximum people capacity in the building in considering the evacuation process.

## 2. Related Works

With the rapid development of sensor technologies and IoT technologies, temperature aware evacuation services are possible in state-of-the-art IoT buildings. Home-based fire detection and alert system prototype were built in [6] by adopting the Arduino board to connect and control temperature sensors, flame sensors and ZigBee technology for transmission module. Another prototype was designed and implemented in [7] to monitor and control the industrial environment by using the Wi-Fi communication module. Hardware solutions, which include RFID platform and temperature sensors, have been designed and implemented in [8] to enable emergency evacuation services in IoT based buildings. In [8], the RFID platform, which includes the RFID tags and RFID readers, is implemented to locate the people in the buildings. Based on the temperature data acquired by the implemented temperature sensors in [8] and the people position information based on the RFID solutions implementation in [8], the central computer can activate the emergency evacuation when a fire is detected. Wireless sensor networks for real-time fire detection are designed and evaluated in [9] to enable fire safety in the home environment. An evacuation support system in large areas is proposed in [10]. The system’s architecture includes the emergency operation centers, localized operation center, smartphone app and wireless backbone networks are described in [10] to show that evacuation support system based on IoT network can adapt to dynamic conditions in the networks and guide the people to the safe places. In [11], they proposed an integrated framework, including the cloud computing center to collect and compute the system information and an indoor localization system based on Bluetooth to locate and evacuate evacuees to the safe zone.

An evacuation route planning algorithm has been proposed in recent studies. In [12,13], a shortest-path-based algorithm is proposed to guide the evacuees to the nearest exit. In [14,15], the evacuation algorithms based on ant colony algorithm is proposed and simulated. The basic idea of the ant colony algorithm is based on the cooperative behavior of the evacuee to search for a safe emergency exit. In [16], the bee colony optimization approach is proposed to find the evacuation path via the idea of swarm intelligence from the bee colony. In [17], the first multi-exit evacuation algorithm is proposed to evacuate crowded people in large buildings. The basic idea in [17] is to partition the evacuees into groups and evacuate each group to its nearest exit. To avoid congestion problems at the exits, the departure time of each group is calculated and delayed in its evacuation sequence. In [11], a risk score based on the weighted sum of risk factors (includes temperature, visibility, CO density, etc.) for each possible evacuation path is calculated to identify the safe evacuation route. In [18], Dijkstra’s shortest path evacuation path algorithm is proposed to identify the best route based on the risk assessment of numerically simulated fire scenarios.

Evacuation in the open space from disaster or terrorist attack has been proposed in [19]. As compared to the building environment, it is not easy to install the sensors in the open space to acquire the data from the environment. Lacking environmental data, the evacuation strategy relies on the evacuee’s personal judgment and group behavior. Such kind of evacuation leveraging on evacuee’s individual intelligence and swarm intelligence also has been applied in high rise building [20].

Evacuee’s behavior is an important factor in the evacuation success rate. In [21], they found out that occupants will not immediately evacuate their residence when they hear a fire alarm because they think it is a drill or false alarm. Interestingly, if the occupants are informed of the fire locations and evacuation path, the reluctance to evacuate is reduced so as to speed-up the evacuation. In [22], they propose an emotional contagion model of the crowded people in the fire. They found out that evacuee’s degree of panic is determined by accident information, evacuee’s knowledge and comfort level. Moreover, accident information can be provided by IoT devices to help reduce the degree of panic. In [23], simulations show that evacuee’s evacuation behavior is seriously affected by the fire. The smoke spreading process and the effects on evacuees will be smaller at the exit that is far from the fire. The effect of environmental knowledge in evacuation has been studied in [24]. The results show that visitors need longer evacuation time than occupants with the knowledge of the building.

Fire and evacuation simulation models have been proposed in recent research. In [25], fire and evacuation software is developed to simulate the evacuation time in various environmental scenarios. Another study in [26] proposed a fire evacuation simulation technique to capture the movement of evacuees with considering the fire spread. In [27], agent-based models that consist of four agents (evacuees, fire, smoke and alarm) are proposed and simulated at supermarkets. When temperature data in the building is not available, the evacuee’s personal decisions will determine the evacuation success rate. In [28], an exit choice model was proposed and simulated by assuming the fire spreading, and the crowdedness of the exits can be predicted by the evacuee. Fire and evacuation simulations done in [29] are used to reconstruct a fatal fire that occurred in an indoor shooting range in Pusan, Korea, in 2009. They found out that occupants’ response time is the most important factor in an evacuation. Computational experiments and simulations are performed in [30] to improve evacuation efficiency in different settings of fire scenarios and evacuee compositions. Fire and evacuation simulations [31] and fire evacuation system [32] were performed at the historical museum [31] and historical theatres [32] in Italy to increase the evacuation efficiency without altering the architectural characteristics of historical buildings.

The fire evacuation problem is a complex and highly dynamic problem. Until now, there are no international standards on the verification and validation of building fire evacuation models [33]. In [33], they suggest five main components of the fire evacuation model should be included, namely evacuation time, movement and navigation, exit usage, route availability and flow constraints. The fire evacuation model in large buildings needs to address more on the building architecture and floor plan that determines the evacuation routes. Artificial neural network (ANN) based fire evacuation algorithm is proposed in [34] to identify the best evacuate route by calculating the risk value on each link. However, fire evacuation planning by ANN algorithm or reinforcement learning algorithm is a challenging problem because there is no standard benchmark environment to train these artificial intelligence agents. The first fire evacuation environment to train reinforcement learning agents for evacuation planning is proposed in [35].

To summarize, even though there are many evacuation algorithms proposed in the literature, most of these algorithms are a one-shot algorithm where they do not address the temperature changing problem in the evacuation. In particular, how to identify a temperature-aware evacuation path in the multi-story multi-exit building is still not explored yet. In this paper, we capture the temperature changes at each time slot and propose a novel mathematical model and algorithms to identify the efficient evacuation path in a multi-story multi-exit building.

## 3. Graph Mapping Scheme from Three-Dimension Building to Two-Dimension Graph

To successfully develop the evacuation path in a multi-floor building, it is important to capture the building’s information in the three-dimension structure at the first stage. The floor plan information (including the sensor locations, doors, walls and obstacles) at each floor must be captured. In addition, the staircase that connects each floor also must be identified. As it is known that it is easier to develop the evacuation path algorithm in a two-dimensional graph. We first develop a graph mapping scheme that transforms the three-dimensional multi-floor building into a two-dimensional graph.

First, let Γ indicates the set of possible locations to deploy a temperature sensor in the multi-floor building. The location (i, j,k) in set Γ indicates the location to deploy a temperature sensor at location (*i*, *j*) on floor *k* where i∈I,j∈J and k∈K. Here, set *I* indicates the set of X-axis locations; set *J* indicates the set of Y-axis locations and set *K* indicates the set of floor locations in the multi-floor building. Then the total number of locations to deploy the sensors is (|I|·|J|·|K|). Next, let δ(i, j,k) indicates the corresponding node ID number at location (*i*, *j*) on floor *k* and δ∈Δ where Δ is the set of nodes on the planar graph. To transform the three-dimensional multi-floor building to the two-dimensional planar graph, each node in Γ(i, j,k) will associate with a node in Δ and this is a one-to-one mapping. Then we have Equation (1),

(1) |I|·|J|·|K|=|Δ|

Next, we assign the node ID number δ(i, j,k). The node ID assignment starts with the top left position Γ(1, 1,1) at the lowest floor in the building and all the way to the bottom-right position Γ(|I|, |J|,|K|) on the highest floor in the building. The mapping is a row-based mapping that after all the positions in row *i* is associated with a node ID, then next move to the positions in row (i+1). Then for any Γ(i, j,k), the node ID δ(i, j,k) is calculated at Equation (2).

(2) δ(i, j,k)=(j+(i−1)×|J|+(k−1)×|I|×|J|)

A staircase is a connection between the upper and lower floor of the building. Let there are two staircases in the building where the location is at the bottom corner of each floor. For the highest floor (i.e., |K|th floor), the two staircase locations are at the bottom left position, and bottom-right position that connects to the bottom left position and bottom-right position at the (|K|−1)th floor, respectively. For the lowest floor (i.e., first floor), the two staircase locations are at the bottom left position and bottom-right position that connects to the bottom left position and bottom-right position at the second floor, respectively. For any *k* floor that is neither the highest floor nor the first floor (i.e., 1<k<|K|), there are also two staircase locations. The two staircase locations are at the bottom left position and bottom-right position that connects to the bottom left position and bottom-right position at the (k+1)th floor, respectively. In addition, these two staircase locations also connect to the bottom left position and bottom-right position at the (k−1)th floor, respectively.

Based on Equation (2) and the above arguments, the node ID number for the left staircase at the αth floor is shown in Equation (3).

(3) δ(|I|, 1,α)=(1+(|I|−1)×|J|+(α−1)×|I|×|J|)

In addition, the node ID number for the right staircase at the αth floor is shown in Equation (4).

(4) δ(|I|,|J|,α)=(|J|+(|I|−1)×|J|+(α−1)×|I|×|J|)

In Figure 1, we illustrate an example of a three-floor building where each floor has a grid size of 10×10. The number with the blue color indicates the node ID in Figure 1. In this case, |I|=10, |J|=10 and |K|=3. In Figure 1, each floor has 100 locations to deploy a temperature sensor. According to Equation (2), the node ID for location (10, 2, 1) on the first floor is (2+(10−1)×10+(1−1)×10×10)=92.

After getting the node ID, the next step is to set up the links to connect the nodes in the planar graph. Basically, for any location in Γ(i, j,k) that is not the staircase location; there is a link to each of four its neighboring locations (i.e., up to the location, down the location, left the location and right location) on the same floor *k*. Let set Φ denote the set of links in the planar graph. Then, ∀ξ∈Φ, we have

(5) $$\mathrm{Setup}\;\mathrm{uplink}\;\xi_{\Gamma \left(i,j,k\right)\Gamma \left(i-1,j,k\right)}\;\;\;\forall i\in I-\left\{1\right\},j\in J,k\in K$$

(6) $$\mathrm{Setup}\;\mathrm{downlink}\;\xi_{\Gamma \left(i,j,k\right)\Gamma \left(i+1,j,k\right)}\;\;\;\forall i\in I-\left\{\left|I\right|\right\},j\in J,k\in K$$

(7) $$\mathrm{Setup}\;\mathrm{left}\;\mathrm{link}\;\xi_{\Gamma \left(i,j,k\right)\Gamma \left(i,j-1,k\right)}\;\;\;\forall i\in I,j\in J-\left\{1\right\},k\in K$$

(8) $$\mathrm{Setup}\;\mathrm{right}\;\mathrm{link}\;\xi_{\Gamma \left(i,j,k\right)\Gamma \left(i,j+1,k\right)}\;\;\;\forall i\in I,j\in J-\left\{\left|J\right|\right\},k\in K$$

The reason why we have ∀i∈I−{1} in (5) is that the locations in the first row do not have the up neighbors. Likewise, ∀i∈I−{|I|} in (6) is because the locations in the last row do not have the down neighbors; ∀j∈J−{1} in (7) is because the locations in the first column do not have the left neighbors; ∀j∈J−{|J|} in (8) is because the locations in the last column do not have the right neighbors. Next, we set up the links to connect the upper location and the lower location at the staircase.

(9) $$\mathrm{Setup}\;\mathrm{vertical}\;\mathrm{uplink}\;\xi_{\Gamma \left(i,j,k\right)\Gamma \left(i,j,k+1\right)}\;\;\;\forall i\in \left\{\left|I\right|\right\},j\in \left\{1,\;\left|J\right|\right\},k\in K-\left\{\left|K\right|\right\}$$

(10) $$\mathrm{Setup}\;\mathrm{vertical}\;\mathrm{downlink}\;\xi_{\Gamma \left(i,j,k\right)\Gamma \left(i,j,k-1\right)}\;\;\;\forall i\in \left\{\left|I\right|\right\},j\in \left\{1,\;\left|J\right|\right\},k\in K-\left\{1\right\}.$$

Based on Equations (5)–(10), the total number of links in the planar graph is calculated in Equation (11). In Equation (11), the first term is to calculate the total links for all four directions of every node. The second term is to deduct the number of links that the nodes do not have their neighbors. The third term is to add the number of links that connect the staircase locations. For the three-floor building example shown in Figure 1, there are 1088 links to be set in the planar graph.

(11) |Φ|=(|I|×|J|×|K|×4)−((|I|×2+|J|×2)×|K|)+(2×2×(|K|−1)).

Based on the Equations (1)–(4) to determine the node ID and Equations (5)–(10) to determine the link ID, we successfully transform the multi-floor building locations to the planar graph. In the next section, we will describe the algorithms to identify the efficient evacuation path in the multi-story multi-exit building.

## 4. Mathematical Model for Evacuation Path Planning in Multi-Story Multi-Exit Building

In this section, we propose the mathematical model to capture the evacuation path planning in the multi-floor building. The set of nodes and links in the planar graph constructed in the previous section are used as the input parameters to the following mathematical models. The basic idea of this mathematical model is to identify the minimum cost evacuation path in the multi-floor building. We generalize the model to identify the cost-efficient evacuation path. The cost can be the number of steps to evacuate from the building or the total temperature along the evacuation path. By setting the cost to be the number of steps to evacuate from the building, it is to minimize the time to evacuate. By setting the cost to be the total temperature along the evacuation path, it is to identify the evacuation path that can avoid the fire zone.

In the following, we first propose the mathematical model to capture the evacuation path planning in the multi-floor building.

### 4.1. Evacuation Path (EP) Model

First, we give the notations used in the formulation as follows:
Input values: U: the set of users; $P_{u}$: the set of evacuation path selected by user u∈U; *L*: the set of possible links on the planar graph; *E*: the set of emergency exit nodes on the planar graph; *M*: the upper limit of human temperature tenability (e.g., 100 °C); $a_{l}$: the link cost on link l∈L; $\tau_{l}$: the temperature on link l∈L; $\delta_{pl}$: the indication function, $\delta_{pl}$ = 1 when the link l is on the evacuation path p; $\delta_{pl}$ = 0, otherwise; $\zeta_{pe}$: the indication function, $\zeta_{pe}$ = 1 when the node e is on the evacuation path p; $\zeta_{pe}$ = 0, otherwise; Decision variables: $x_{p}$*:* =1 if evacuation path *p* is selected where $p\in P_{u},u\in U$; =0, otherwise; $y_{l}$*:* =1 if link *l* is on the selected evacuation path where l∈L*;* =0, otherwise.

The EP mathematical model formulation is proposed as follows:

Problem (P_1_):

$$Z_{p1}\;=\;\min\;\sum_{l\in L}a_{l}\times y_{l}$$

Subject to:

(12) $$x_{p}\times \delta_{pl}\leq y_{l}\;\;\forall p\in P_{u},u\in U,l\in L$$

(13) $$y_{l}\times \tau_{l}\leq M\;\;\forall l\in L$$

(14) $$\underset{p\in P_{u}}{\sum }x_{p}=1\;\;\forall u\in U$$

(15) $$\underset{p\in P_{u}}{\sum }\underset{e\in E}{\sum }x_{p}\zeta_{pe}\geq 1\;\;\forall u\in U$$

(16) $$x_{p}\;=\;0\;\mathrm{or}\;1\;\;\forall p\in P_{u},u\in U$$

(17) $$y_{l}\;=\;0\;\mathrm{or}\;1\;\forall l\in L.$$

In problem (P_1_), the objective function is to minimize the total cost on the evacuation path. Constraints (14) and (16) enforce that there is an evacuation path for every user u∈U. Constraints (12) and (16) enforce that$\;y_{l}$ = 1 when link *l* is on the evacuation path. Constraint (13) enforces for the temperature on any selected link cannot exceed the upper limit of human temperature tenability *M*. This constraint is very important that people can survive on the evacuation path. Without this constraint, people may be hurt or even die on the evacuation path. Constraint (15) enforces that the evacuation path should traverse through at least one evacuation exit node. This is to ensure that every user can find the evacuation path to the emergency exit. Note that if the evacuation path for any user traverses through more than one emergency exit node, the traverse route after the first emergency exit node can be deleted to have a lower objective function value without violating the constraints in problem (P_1_). Hence, at the optimal solution, each user will traverse only one emergency exit.

In problem (P_1_), when setting $a_{l}$ = 1 ∀l∈L*,* the objective function is to minimize the number of steps to reach the destination (i.e., emergency exit). By adopting this link cost setting, it is to identify the fastest evacuation path to reach the destination. When $a_{l}$ = $\tau_{l}$ ∀l∈L*,* the objective function becomes to minimize the total temperature on the evacuation path to reach the destination. By adopting this link cost setting, it is to identify the lowest temperature evacuation path to reach the destination. In other words, the proposed TAEP model can be used to identify the fastest evacuation path model or the lowest temperature evacuation path model by changing the link cost setting.

However, this EP model is only valid to a slow fire progression area or a small area where the people can run to the emergency exit quickly without the temperature changes on the evacuation path. In a multi-floor building, the people need minutes of evacuation time to run to the emergency exit. In this case, the temperature on the evacuation path may significantly increase so that people may be stuck at the evacuation path because the temperature is already over the upper limit of human temperature tenability *M*. To be more specific, EP is a mathematical model to identify the evacuation path by assuming the fixed temperature on the evacuation path, which is not valid in a large area (e.g., multi-floor building).

Next, we consider the mathematical model to address the temperature changes during evacuation in the multi-floor building. The basic idea is that the temperature sensors will report the temperature in a fixed interval (e.g., one minute) so that the link cost will be adjusted to address the temperature changes at the link’s termination node.

### 4.2. Temperature and Time-Aware Evacuation Path (TTAEP) Model

Besides the notations given in Section 4.1, the additional notations used in the TTAEP model are listed as follows:
Input values: *T*: the set of time slots during evacuation; $\sigma_{lt}$: the temperature on link l∈L at time slot *t*
∈T; $b_{lt}$: the link cost on link l∈L at time slot *t*
∈T; Decision variables: $z_{lt}$*:* =1 if link *l*
∈L is on the selected evacuation path at time slot *t*
∈T*;* =0, otherwise.

The TTAEP mathematical model formulation is proposed as follows:

Problem (P_2_):

(18) $$Z_{p2}=\;\min\;\sum_{l\in L}\sum_{t\in T}b_{lt}\times z_{lt}$$

Subject to:

(19) $$y_{l}\leq \underset{t\in T}{\sum }z_{lt}\;\forall l\in L,t\in T$$

(20) $$x_{p}\times \delta_{pl}\leq y_{l}\;\;\forall p\in P_{u},u\in U,l\in L$$

(21) $$z_{lt}\times \sigma_{lt}\leq M\;\;\forall l\in L,t\in T$$

(22) $$\underset{p\in P_{u}}{\sum }x_{p}=1\;\;\forall u\in U$$

(23) $$\underset{p\in P_{u}}{\sum }\underset{e\in E}{\sum }x_{p}\zeta_{pe}\geq 1\;\;\forall u\in U$$

(24) $$\underset{t\in T}{\sum }z_{lt}\leq 1\;\;\forall l\in L$$

(25) $$x_{p}=0\;\mathrm{or}\;1\;\;\forall p\in P_{u},u\in U$$

(26) $$y_{l}=\;0\;\mathrm{or}\;1\;\;\forall l\in L$$

(27) $$z_{lt}\;=\;0\;\mathrm{or}\;1\;\;\forall l\in L,t\in T.$$

In problem (P_2_), the objective function is to minimize the total cost in addressing the time aware temperature changes on the evacuation path. The reason why we introduce the $b_{lt}$ and $z_{lt}$ with respect to the time slot, *t* ∈T on link l∈L is that the temperature may be increasing rapidly in the fire zone. In a large multi-floor building, the evacuation time is long enough that the temperature change along the evacuation path is non-negligible. Hence, the link cost should be adjusted according to the temperature changes along the evacuation path. By assuming that sensors periodically report the temperature back to the sink node, the link cost can incorporate the temperature changes at each node. With introducing the variable, $b_{lt}$, the current temperature at time slot *t* ∈T on link l∈L is captured in the link cost on the planar graph. In addition, with introducing the decision variable, $z_{lt}$, the link selection on the evacuation path addresses the most recent temperature data at time slot *t* ∈T on link l∈L.

In problem (P_2_), constraints (22) and (25) enforce that there is an evacuation path for every user u∈U. Constraints (20) and (26) enforce that$\;y_{l}\;$= 1 when link *l* is on the evacuation path. Constraint (21) enforces that when people traverse link l∈L at time slot *t* ∈T, the associated temperature cannot exceed the upper limit of human temperature tenability *M*. Constraints (19) and (27) enforce that for any link *l* ∈T, if link *l* is on the evacuation path, people must traverse this link at least one time slot (i.e.,$\;\sum_{t\in T}z_{lt}\geq \;$1) during evacuation. Then, the objective function can add all the time-aware link costs on the evacuation path and identifies the minimum time-aware cost evacuation path. Even though the objective function and the above constraints capture the idea of identifying evacuation path in considering the temperature changes in different time slots, we put constraint (24) in problem (P2) to restrict the space of feasible solutions without sacrificing the optimality.

Constraint (24) is to enforce that people can only traverse every link l∈L on the evacuation path at most one time slot. Recall that in constraint (19), people must traverse every link on the evacuation path at least one time (i.e.,$\;\sum_{t\in T}z_{lt}\geq$1). Then combining constraint (19) and constraint (24), it becomes to enforce that people traverse every link l∈L on the evacuation path exactly one time. It is straightforward that at the optimal solution, every link l∈L on the evacuation path can only be traversed one time. If a link is traversed more than one time, then there is a loop on the evacuation path. By deleting the loop from the evacuation path, the remaining evacuation path is still a path from the source to the destination with a more cost-efficient solution. Hence, this justifies that add constraint (24) to problem (P2) can restrict the space of feasible solutions without sacrificing the optimality.

In the next section, we propose the algorithms to solve the TTAEP mathematical models.

## 5. Solution Approaches

Problems (P_2_) contains a constrained shortest path problem, and it is proven to be an NP-hard problem in [36]. Hence, there is no polynomial-time algorithm to optimally solve problems (P_2_). We propose two sets of algorithms to tackle this multi-floor evacuation path problem. The first set is the three shortest path-based algorithms without addressing the temperature constraint (21). In this first set, the first algorithm is the shortest path (SP), which is leveraged on Dijkstra’s shortest path algorithm. The second algorithm is the best-first search (BFS) that is leverage on the A* algorithm. The third algorithm is dynamic best-first search (DBFS) that is leverage on the D* algorithm. The second set is the three shortest path-based algorithms with addressing the temperature constraint in (21). They are temperature aware best-first search (TABFS), temperature aware shortest path (TASP) and temperature aware dynamic best-first search (TADBFS) algorithms. Note that only the three algorithms in the second set (i.e., TABFS, TASP, TADBFS) identify the feasible solutions to the TTAEP problem in problem (P2), which can be used to evacuate the evacuees. Three algorithms (BFS, SP, DBFS) that do not address the human’s temperature limit constraint can be used by the rescue robots or fireman with a fire-proof suit.

### 5.1. Three Shortest Path-Based Algorithms without Addressing the Temperature Constraint (21)

In the following, we first present the SP algorithm (Algorithm 1). The basic idea of the SP algorithm is to leverage Dijkstra’s algorithm to identify the shortest path to every exit based on the temperature data acquired by sensors at each time slot *t*. Then among these shortest paths to every exit, identify the exit (say $\tilde{\lambda }$) with the minimum path cost and let the best path to exit $\tilde{\lambda }$ be the evacuation path at this time slot. Considering the number of hops a user can move is limited in one time slot, a user should traverse the evacuation path as much as possible towards the exit $\tilde{\lambda }$. Let Λ be the number of steps a user can, at most, move in the period of one time slot. If the hop distance between the user’s current position and exit $\tilde{\lambda }$ is not greater than Λ, the user can move to the emergency exit $\tilde{\lambda }$ and report the total cost along the evacuation path. Otherwise, the user moves Λ hops on this evacuation path towards the exit $\tilde{\lambda }$ and the algorithm repeats until the user reaches an emergency exit.
**Algorithm 1: SP Algorithm****Begin** **Initialize** the value of all the decision variables to be zero; **Construct** the nodes and links in the 2-D planar graph; **Let** the node position of the evacuating person be the start node; **While**
(t≤|T|) //looping at each time slot *t* **Begin** **Collect** the temperature data at each node and the link cost data at each link at time slot t; **Calculate** the best path from the start node to every exit node by using Dijkstra’s algorithm and let $\lambda_{e}=$ the shortest path cost to every exit e∈E; //Step 2 **Let**
$\tilde{\lambda }=\mathrm{Min}\;Arg_{e\in E}\lambda_{e}$; //the exit number that has the smallest shortest path cost, evacuation path is the best path to exit $\tilde{\lambda }$ **If** (the hop distance from the start node to the exit number $\tilde{\lambda }$ on the shortest path ≤
Λ) //reach the exit **Begin**  **Let**
Ω = Ω + (The link cost from the start node to the exit $\tilde{\lambda }$ on this evacuation path);  **Report**
Ω and the evacuation path;  ρ = 1;  **Break** from the **While** loop; **End** // If (the hop **Else** // have not reached the exit, then move the start node Λ hop distance closer to exit $\tilde{\lambda }$ on the emergency path **Begin**  **Let**
Ω = Ω + (All the link cost from the start node to the node with hop distance Λ on the evacuation path);  **Move** the start node to the new position with Λ hop distance on the evacuation path; **End** // Else **End**; //While loop **If** (ρ == 0)  **Report** no feasible solutions; **End**

In the SP algorithm, the time complexity is determined by the “while” loop for each time slot. Inside the “while” loop, Dijkstra’s shortest path algorithm must be performed for every emergency exit. The time complexity of Dijkstra’s shortest path algorithm is $O\left({\left|N\right|}^{2}\right)$ where *N* is the set of nodes in the networks. Then the time complexity for the SP algorithm is $O\left(\left|T\right|\times \left|E\right|\times {\left|N\right|}^{2}\right)$, where |T| is the maximum number of time slots and |E| is the maximum number of exits in the networks.

The second algorithm is the BFS algorithm. By changing Dijkstra’s shortest path algorithm to A* algorithm at Step 2 in the “while” loop, the above algorithm becomes the BFS algorithm. The basic idea of the BFS algorithm is to leverage on A* algorithm [37] to identify the best path to every exit based on the temperature data acquired by sensors at each time slot *t*. The A* algorithm is an iteration-based algorithm to identify the shortest path from the start node to the destination node. At each iteration of extending the path to the destination node, A* identifies the next node n that minimize the *f*(*n*) = *g*(*n*) + *h*(*n*), where *g*(*n*) is the cost from the start node-to-node *n* and *h*(*n*) is the heuristic function that estimates the cost from node *n* to the destination node [37]. Here we set *h*(*n*) to be the Euclidean distance from node *n* to the destination node. Because the Euclidean distance is smaller than the actual distance, the A* algorithm can identify the shortest path from the source node to the destination node.

In the BFS algorithm, the time complexity is determined by the “while” loop for each time slot. Inside the “while” loop, A* shortest path algorithm must be performed for every emergency exit. When using the binary heap data structure, the time complexity of A* shortest path algorithm is O(|N|log|N|) where *N* is the set of nodes in the networks [38]. Then the time complexity for the BFS algorithm is O(|T|×|E|×|N|log|N|), where |T| is the maximum number of time slots and |E| is the maximum number of exits in the networks.

The third algorithm is the DBFS algorithm. By changing Dijkstra’s shortest path algorithm to D* algorithm at Step 2 in the “while” loop, the above algorithm becomes the DBFS algorithm. The basic idea of the DBFS algorithm is to leverage on D* algorithm [39] to identify the best path to every exit based on the temperature data acquired by sensors at each time slot *t*. In the DBFS algorithm, the time complexity is determined by the “while” loop for each time slot. Inside the “while” loop, D* shortest path algorithm must be performed for every emergency exit. The time complexity of D* shortest path algorithm is O(|N|log|N|), which is the same as the time complexity of the A* algorithm [38,39]. Then the time complexity for the DBFS algorithm is O(|T|×|E|×|N|log|N|), where |T| is the maximum number of time slots and |E| is the maximum number of exits in the networks.

### 5.2. Three Shortest Path-Based Algorithms with Addressing the Temperature Constraint (21)

First, we show the TASP algorithm (Algorithm 2). As compared to the SP algorithm, the link temperature that is over the upper limit of human temperature tenability *M* is removed from the set *L* and then perform Dijkstra’s algorithm to identify the shortest evacuation path. With this operation, the temperature on all the links selected by the identified evacuation path will not exceed the upper limit of human temperature tenability *M*. Hence, constraint (21) is captured and satisfied.
**Algorithm 2: TASP Algorithm****Begin** **Initialize** the value of all the decision variables to be zero, and the node position of the evacuating person be the start node;  **Construct** the nodes and links in the 2-D planar graph;  **Let** the node position of the evacuating person be the start node;  **While**
(t≤|T|) //looping at each time slot *t*  **Begin** **Collect** the temperature data at each node and the link cost data at each link at time slot t; **Remove** the link *L* from set *L* if the link temperature is over the upper limit of human temperature tenability *M*; //avoiding traverse to a node with temperature more than *M* on the evacuation path **Calculate** the shortest path from the start node to every exit node by using Dijkstra’s algorithm; //Step 3 **Let**
σ = 0; **For** every exit e∈E **Begin** **If** there is the best path to exit e∈E **Begin** **Let**
$\lambda_{e}=$ the best path cost to exit e∈E; σ = 1; **End** //if **End** //For   **If** (σ == 0)//cannot find any best path to all the exits   **Begin**    **Report** no feasible solutions;   **Break** from the **While** loop;  **End** //If (σ == 0)  **Else** //at least there is the shortest path to exit **Let**
$\tilde{\lambda }=\mathrm{Min}\;Arg_{e\in E}\lambda_{e}$; //the exit number that has the smallest shortest path cost, evacuation path is the best path to exit $\tilde{\lambda }$ **If** (the hop distance from the start node to the exit number $\tilde{\lambda }$ on the evacuation path ≤
Λ) //reach the exit **Begin** **Let**
Ω = Ω + (The link cost from the start node to the exit $\tilde{\lambda }$ on the evacuation path); **Report**
Ω and the evacuation path; ρ = 1; **Break** from the **While** loop; **End** // If (the hop **Else** // have not reached the exit, then move the start node Λ hop distance closer to exit $\tilde{\lambda }$ on the emergency path **Begin** **Let**
Ω = Ω + (All the link cost from the start node to the node with hop distance Λ on the evacuation path); **Move** the start node to the new position with Λ hop distance on the evacuation path; **End** // Else **End**;//While loop **If** (ρ == 0)  **Report** no feasible solutions; **End**

In the above TASP algorithm, the time complexity is also determined by the “while” loop for each time slot. Inside the “while” loop, Dijkstra’s shortest path algorithm must be performed for every emergency exit. The time complexity of Dijkstra’s shortest path algorithm is $O\left({\left|N\right|}^{2}\right)$ where *N* is the set of nodes in the networks. Then the time complexity for the TASP algorithm is $O\left(\left|T\right|\times \left|E\right|\times {\left|N\right|}^{2}\right)$, where |T| is the maximum number of time slots and |E| is the maximum number of exits in the networks. A* algorithm must be performed for every emergency exit. The time complexity of the A* algorithm is O(|L|) because A* algorithm needs to travel all the links to reach the destination in the worst case. Then the time complexity for the BFS algorithm is O(|T|×|E|×|L|), where |T| is the maximum number of time slots and |E| is the maximum number of exits in the networks.

The next algorithm is the TABFS algorithm. By changing Dijkstra’s shortest path algorithm to A* algorithm at Step 3 in the “while” loop, the above algorithm becomes the TABFS algorithm. In the TABFS algorithm, the time complexity is also determined by the “while” loop for each time slot. Inside the “while” loop, the A* algorithm must be performed for every emergency exit. The time complexity of the A* algorithm is O(|N|log|N|) where *N* is the set of nodes in the networks [38]. Then the time complexity for the BFS algorithm is O(|T|×|E|×|N|log|N|), where |T| is the maximum number of time slots and |E| is the maximum number of exits in the networks.

The next algorithm is the TADBFS algorithm. By changing Dijkstra’s shortest path algorithm to the D* algorithm at Step 3 in the “while” loop, the above algorithm becomes the TADBFS algorithm. In the TADBFS algorithm, the time complexity is also determined by the “while” loop for each time slot. Inside the “while” loop, D* algorithm must be performed for every emergency exit. The time complexity of the D* algorithm is O(|N|log|N|) where *N* is the set of nodes in the networks [38,39]. Then the time complexity for the TADBFS algorithm is O(|T|×|E|×|N|log|N|), where |T| is the maximum number of time slots and |E| is the maximum number of exits in the networks.

To summarize, the basic idea of the proposed algorithms is to identify the best path based on the temperature data at the current time slot and the evacuee move Λ hop distance along the path. If the evacuee has not reached the destination, the proposed algorithms execute again, and the evacuee move Λ hop distance along the new evacuation path based on the temperature data at the next time slot. This process keeps repeating until either the evacuee reaches the emergency exit (i.e., evacuation success) or all the emergency exits are on fire (i.e., evacuation failure). Among these six proposed algorithms, SP and TASP are shortest-path-based algorithms by leveraging on Dijkstra’s algorithm; BFS and TABFS are best-path-based algorithms by leveraging on the A* algorithm; DBFS and TADBFS are dynamic best-path-based algorithms by leveraging on the D* algorithm. Three algorithms (BFS, SP, DBFS) do not address the constraint (21) so that the temperature for nodes on the evacuation path may exceed the upper limit of human temperature tenability *M*. These three algorithms can be used by the rescue robots or fireman with a fire-proof suit. The other algorithms (TABFS, TASP, TADBFS) do address the constraint (21) so that the people can move safely on the evacuation path. These three algorithms can be used by the evacuees. For the computational experiments in the next Section, we are particularly interested in the results from TABFS, TASP and TADBFS because they can identify the feasible solutions to problem (P_2_).

## 6. Computational Experiments

To verify the solution quality of the proposed six algorithms, we perform computational experiments on two networks. The first network is an obstacle-free and wall-free building, and the second network is a big shopping mall building with walking paths and walls. In an obstacle-free and wall-free building, the user can move to the neighboring node in four directions (up, down, right and left) if the user’s position is not at the edge of the graph. In a building with walking paths and walls, users can only move on the walking path and cannot go through the wall. The experiments on the first network are to verify the solution quality of these six algorithms of evacuating people in the open space building (e.g., car parking building). The experiments on the second network are to verify the solution quality of these six algorithms of evacuating people in a building with many rooms (e.g., shopping mall). In these two sets of experiments, the upper limit of human temperature tenability *M* is set to 100 °C.

Basically, the fire spreading grows like a diamond shape, as shown in Figure 1. In the beginning, the temperature is set to 25 °C at each node. At the beginning of the fire, the temperature at the point of origin will set to 100 °C. At each time slot, the fire will spread to its four neighboring nodes with a one-hop distance. The temperature at these four neighboring nodes with fire will increase 50 °C at each time slot. When the temperature of a node is more than 100 °C, the temperature for this node will increase by 40 °C in the next time slot. If the temperature of a node is bigger than 25 °C but smaller than 100 °C, the temperature of its four neighboring nodes will increase by 10 °C.

**Figure 2:**
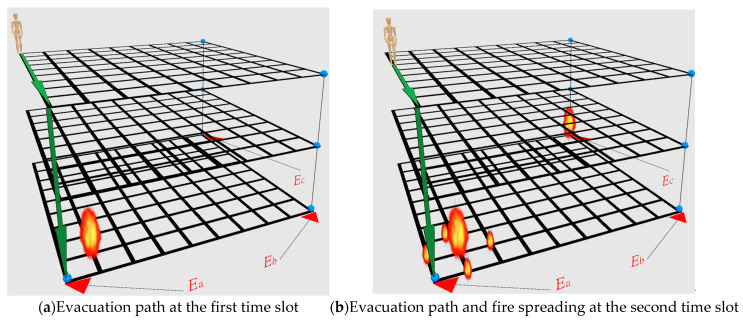
Evacuation in a three-story three-exit building.

We first show the open space network with obstacle-free and wall-free in Figure 2. In Figure 2, there are three emergency exits, and all of these emergency exits are located on the first floor. On the second floor and the third floor, there are also three emergency exits. There is a staircase connecting emergency exits between the upper floor and lower floor. All the emergency exits are colored in blue. A link that connects the emergency exits at the upper layer and the lower layer is the staircase. The user’s position starts at the upper left corner node on the third floor.

In Figure 2, there are two fires in the building. In Figure 2a, the first fire starts at the first time slot is very close to the emergency exit **E_a_** on the first floor. In Figure 2b, the second fire starts at the second time slot is at the emergency exit **E_c_** on the first floor. In Figure 2b, we also observe that the fire spreading to four neighboring nodes at the bottom left corner on the first floor. The hop distance in one time slot is set to three (i.e., Λ = 3). In the first and second time slot, the evacuation path is directed to the emergency exit **E_a_** on the first floor. Because fire spreads one hop distance at each time slot, it can be expected that the fire will spread to the emergency exit **E_a_** at the third time slot. In this case, the temperature for two emergency exits (i.e., **E_a_** and **E_c_**) are all over threshold *M*. Instead of going down the staircase to the emergency exit **E_a_** on the first floor, the evacuation path should be re-directed to the emergency exit **E_b_** on the first floor. In other words, in this scenario, we want to know how well these six algorithms can adapt to the temperature changes in different time slots.

**Figure 3:**
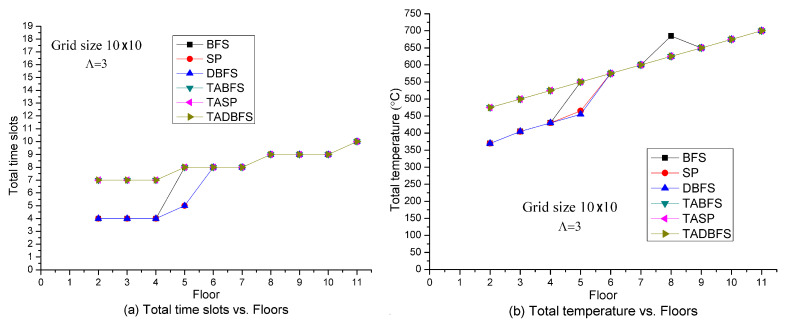
Solution quality comparisons w.r.t. the number of floors in the building.

Figure 3 shows the solution quality of these six algorithms in terms of the total number of time slots and total temperature with respect to the number of floors in the open space building. Note that the initial position of the people is at the upper left node on the highest floor. Since the three safe exits are located on the first floor, the total number of time slots and total temperature needed for the people to evacuate is an increasing function with respect to the number of floors in the building. When the objective function is to minimize the total number of time slots on the evacuation path, the algorithms are to evacuate the people as fast as possible. The results are shown in Figure 3a. In Figure 3b, we set the cost to be the node’s temperature. With this setting, to minimize the total cost on the evacuation path is to minimize the total temperature on the evacuation path. In other words, the objective is to identify an evacuation path with the lowest temperature.

In Figure 3a, we observe that SP, BFS and DBFS algorithms direct the evacuation path to emergency exit **E_a_** in Figure 2 so that the evacuee can evacuate within four time slots when at the lower floor (≤4). On the other hand, TASP, TABFS and TADBFS need seven time slots to evacuate because the evacuation path directs to emergency exit **E_b_** due to the human’s temperature limit constraint in constraint (21). Similarly, we also observe that the total temperature on the evacuation path for SP, BFS and DBFS are much lower than the total temperature on the evacuation path for TASP, TABFS and TADBFS when the number of floors is less than five in Figure 3b. In Figure 3a,b, we also observe that TASP, TABFS and TADBFS have the same evacuation time slots and temperature on the evacuation path.

**Figure 4:**
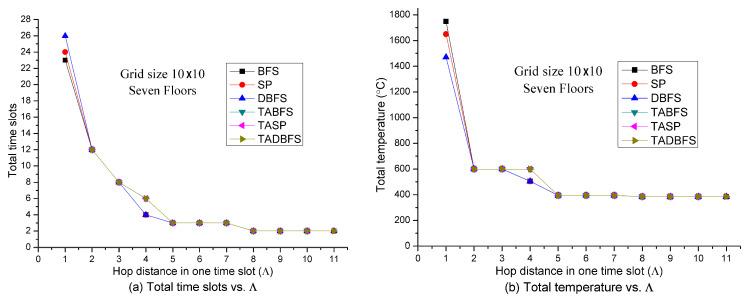
Solution quality comparisons w.r.t. hop distance in one time slot.

Figure 4 shows the solution quality of these six algorithms with respect to the number of hops in one time slot (i.e., Λ). The larger value of Λ, the larger the hop distance evacuee, can move in one time slot. Because the fire spreads one hop in four directions in one time slot, the larger value of Λ, the faster the evacuation speed. It can be expected that the faster evacuation speed will have a smaller number of time slots and total temperature on the evacuation path. The above argument is justified in Figure 4, where the total number of time slots and total temperature on the evacuation path is a decreasing function with respect to Λ. In Figure 4a,b, we observe that TABFS, TASP and TADBFS cannot identify a feasible solution when Λ<2. When Λ=1, the fire spreading speed is faster than the evacuation speed, and the temperature at the exits are all higher than the human’s temperature limit. In Figure 4, we also observe that TABFS, TASP and TADBFS all identify the same evacuation path.

**Figure 5:**
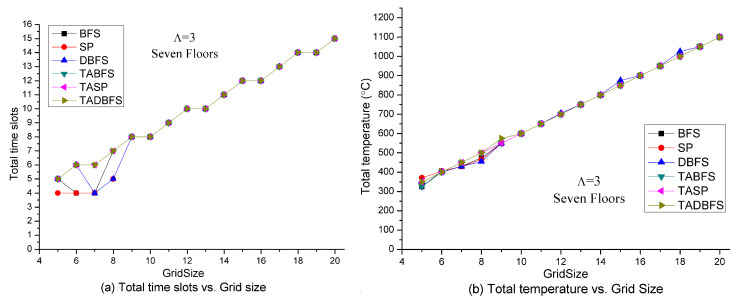
Solution quality comparisons w.r.t. grid size (sensor density).

Figure 5 shows the solution quality of these six algorithms with respect to the grid size. Grid size indicates how many temperature sensors are deployed on each floor. For example, there are 144 temperature sensors deployed at each floor in a 12 × 12 grid size. Increasing the grid size also increases the sensor density. By fixing Λ=3, we can expect that an evacuee needs more evacuation time at a large grid size. Hence, the total number of time slots and total temperature on the evacuation path is an increasing function with respect to the grid size. In Figure 5a,b, we observe that TABFS, TASP and TADBFS all identify the same evacuation path in different sensor density settings. BFS, SP and DBFS identify slightly lower total time slots and total temperature than the TABFS, TASP and TADBFS when the grid size is less than 8 × 8. This shows that human’s temperature limit constraint-in-constraint (21) plays an un-negligible role in determining the evacuation path for TABFS, TASP and TADBFS.

**Figure 6:**
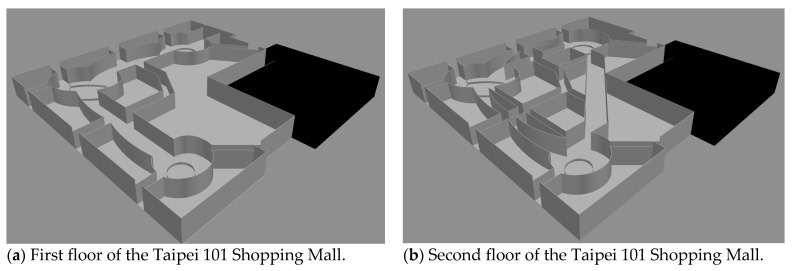
Floor plan of the Taipei 101 Shopping Mall.

Based on the results in Figure 3, Figure 4 and Figure 5, we conclude that TABFS and TASP and TADBFS identify the same evacuation path in the open space building. In the following, we test the solution quality in the building with walking paths and walls. We choose the three-story Taipei 101 Shopping Mall as the network because it is one of the most famous buildings in the world. Figure 6 shows the floor plan in the Taipei 101 Shopping Mall. The floor plan on the third floor, which is not shown in Figure 6, is the same as the first floor. In this three-story shopping mall, there are eight emergency exits on each floor, and there is a staircase connecting every emergency exit between the upper floor and the lower floor.

To capture the walls and walking paths at the Taipei 101 Shopping Mall, a graph mapping scheme in Section 3 is performed to transform the three-dimensional multi-floor building into a two-dimensional graph. The wall is depicted as a sensor node with a very high-temperature so that people cannot go through the planar graph. Note that the graph mapping scheme from the three-dimension building in Section 3 determines the structure of the two-dimension graph. Based on the layout of the three-dimension building and graph mapping scheme, the layout of the two-dimension graph can be any structure. Even though the two-dimension graph is a grid structure in the following computational experiments, the proposed algorithms in Section 4 can tackle any irregular two-dimension graph which is transformed from the three-dimension building.

**Figure 7:**
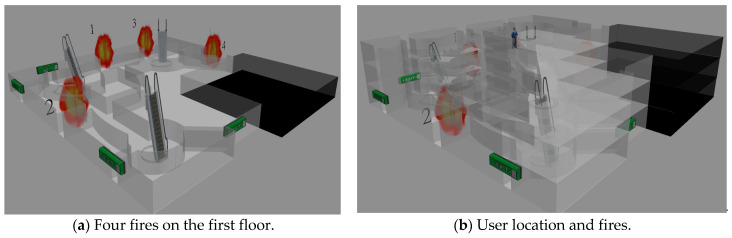
Floor plan and four fires in the Taipei 101 Shopping Mall.

We first test the case when there are four fires on the first floor in the Taipei 101 Shopping Mall. These four fires are located at the four emergency exits on the first floor, which is shown in Figure 7a. The reason why we test four fires is that there are eight emergency exits; we want to test the solution quality of these six algorithms when half of the exits are on fire. The starting position of the evacuee is at the center on the third floor, which is shown in Figure 7b. At the first time slot, there are two fires at emergency exits 1 and 2. At the second time slot, there are two fires at emergency exits 3 and 4.

**Figure 8:**
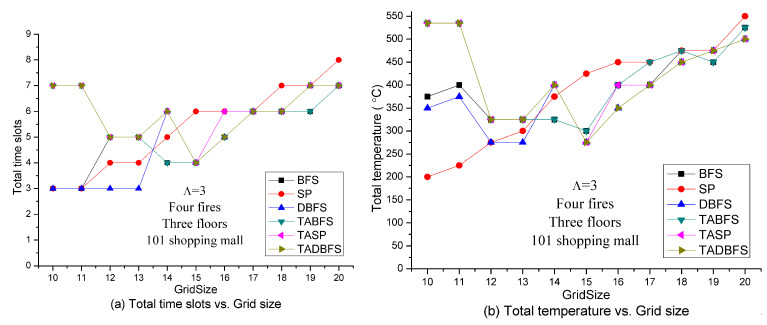
Solution quality comparisons w.r.t. sensor density in the Taipei 101 Shopping Mall with 4 fires.

Figure 8 shows the solution quality of these six algorithms with respect to the grid size. Like the experiments in Figure 5, we can expect the total number of time slots and total temperature needed for the evacuee to evacuate is an increasing function with respect to the grid size. However, there is an interesting result for TASP and TABFS in Figure 8. Grid size 15 × 15 has the best results, both in terms of total time slots and total temperature, for TASP, TABFS and TADBFS in Figure 8a,b. The lowest sensor density at grid size 10 × 10 and highest sensor density at grid size 20 × 20 have poor results, both in terms of total time slots and total temperature, for TASP, TABFS and TADBFS in Figure 8a,b. The results are like a U shape for TASP, TABFS and TADBFS in Figure 8a,b. In the next experiments, we want to test if this result holds seven fires.

**Figure 9:**
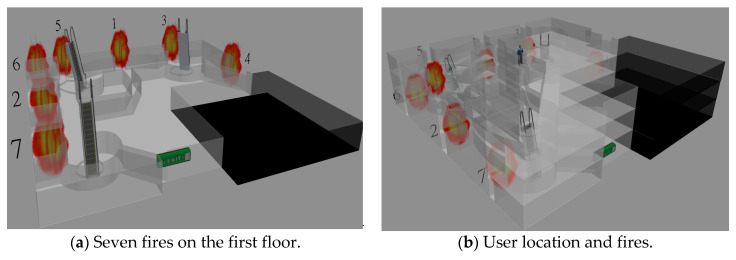
Floor plan and seven fires in the Taipei 101 Shopping Mall.

In the next tested case, we consider there are seven fires on the first floor in the Taipei 101 Shopping Mall. These seven fires are exacted located at the seven emergency exits on the first floor, and there is only one emergency exit that does not have a fire, as shown in Figure 9a. The starting position of people is at the center on the third floor, which is shown in Figure 9b. At the first time slot, there are two fires at emergency exits 1 and 2. At the second time slot, there are two fires at emergency exits 3 and 4. At the third time slot, there are two fires at emergency exits 5 and 6. At the fourth time slot, there is one fire at emergency exit 7. Then, there is only one safe emergency exit left for evacuation.

**Figure 10:**
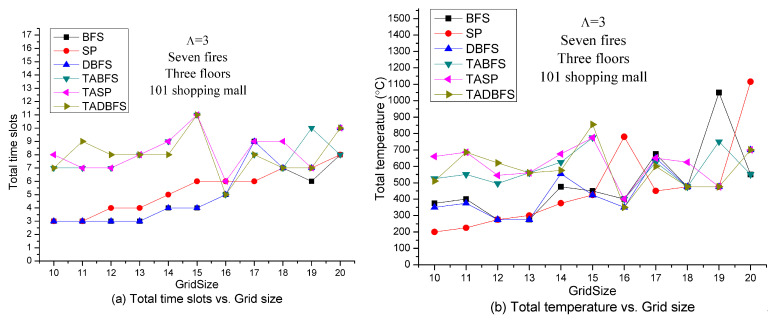
Solution quality comparisons w.r.t. sensor density in 101 shopping mall with 7 fires.

Figure 10 shows the solution quality of these six algorithms with respect to the grid size. Like the results in Figure 8, we also observe a U shape for TASP, TABFS, and TABFS in Figure 10a,b where the best sensor density is at grid size 16 × 16 in terms of time slots and total temperature on the evacuation path. In other words, too low or too high sensor density will increase the evacuation time and temperature on the evacuation path. These results show that the proposed algorithms (TASP, TABFS and TABFS) can help to determine the best temperature sensor deployment density in the Taipei 101 Shopping Mall in considering the evacuee’s evacuation time and evacuation cost.

**Figure 11:**
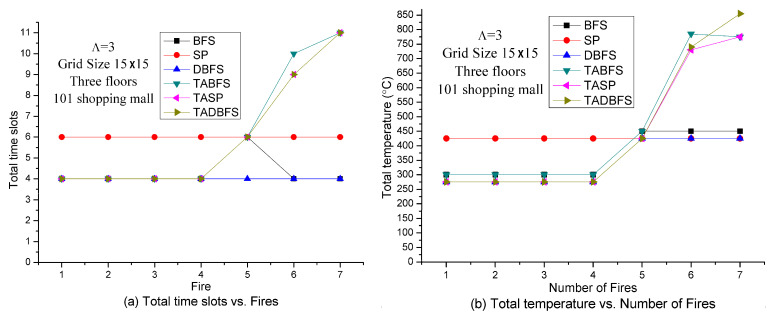
Solution quality comparisons w.r.t. the number of fires in the Taipei 101 Shopping Mall.

Based on the sensor deployment density experiments in Figure 8 and Figure 10, in the next set of experiments, we set the sensor density to be grid size 15 × 15. Figure 11 shows the solution quality comparisons with respect to the number of fires on the emergency exits. It can be expected that the larger number of fires, the lesser number of available emergency exits for evacuation. Because BFS, SP and DBFS do not address the constraint (21), the results are all the same regardless of the number of fires. On the other hand, the solution space for TABFS, TASP and TADBFS becomes smaller when increasing the number of fires on the emergency exit. These three algorithms get almost the same results in evacuation time slots. However, TASP and TADBFS can get better results than TABFS in total temperature on the evacuation path when the number of fires is less than seven. When there are seven fires, TABFS and TASP identify a more comfortable (i.e., lower total temperature) evacuation path than TADBFS.

**Figure 12:**
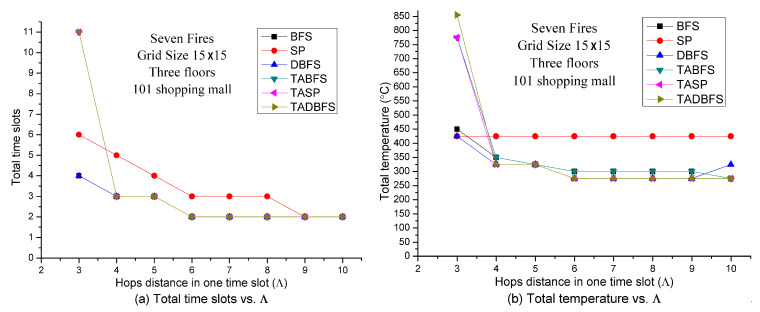
Solution quality comparisons w.r.t. hop distance in one time slot in the Taipei 101 Shopping Mall.

Figure 12 shows the solution quality comparisons with respect to the number of hops in one time slot in seven fires and 15 × 15 grid size. Because the grid size is fixed and the number of fires is also fixed, increasing the value of Λ indicates increasing the evacuation speed. It can be expected that increasing the evacuation speed can increase the success probability of evacuation. Hence, evacuation time and total temperature on the evacuation path should be a decreasing function with respect to the value of Λ. The results in Figure 12 justify the above argument. TABFS, TASP and TADBFS get the same results in total time slots in different values of Λ. However, TASP and TADBFS can get a little bit better results than TABFS in terms of total temperature on the evacuation path when 6≤Λ≤9. When Λ=3, TASP and TABFS can get better results than TADBFS in terms of total temperature on the evacuation path.

**Figure 13:**
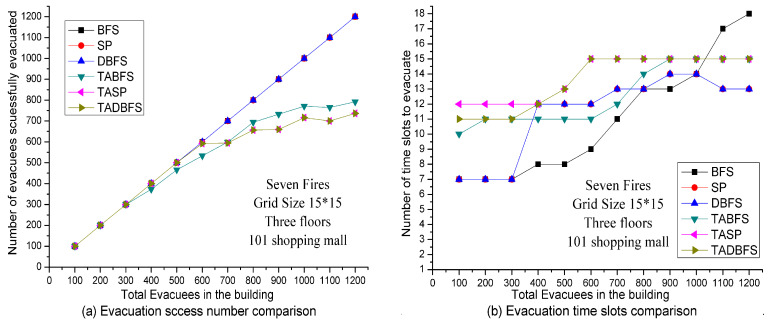
Comparison of evacuation capacity and evacuation speed in the Taipei 101 Shopping Mall.

In the above experiments, the solution quality comparison between these six algorithms is examined for a single evacuee. In the next experiments, we test the solution quality of six algorithms for a group of evacuees. One difference between evacuating a single evacuee and evacuating a large number of people in the evacuation capability in one time slot. The other difference is the computational time of the evacuation algorithm is required to be faster than the fire spreading speed when evacuating a group of people. In Figure 13, we compare the solution quality in terms of evacuation capacity and evacuation speed in the Taipei 101 Shopping Mall. Here, we also set the sensor density to be grid size 15 × 15 on each floor. When evacuating a group of people, the number of people who can pass through an emergency exit at each time slot is limited by the evacuation capability. In the following experiments, we set at most thirty evacuees that can pass through an emergency exit in one time slot. Hence, if more than thirty evacuees evacuate at the same emergency exit at the same time slot, then only 30 evacuees can be evacuated, and the other evacuees should wait to be evacuated at the next time slot.

In Figure 13a, the number of evacuees successfully evacuated is compared between these six algorithms. Because BFS, SP and DBFS do not address the human’s temperature limit constraint in constraint (21), all the evacuees in the building can be evacuated eventually. Hence, the number of evacuees successfully evacuated is the same as the number of evacuees in the building. On the other hand, the number of evacuees successfully evacuated is less than or equal to the number of evacuees in the building for TABFS, TASP and TADBFS algorithms because of human’s temperature limit constraint in Constraint (21). Interestingly, we observe that TASP and TADBFS have the same number of evacuees successfully evacuated. TASP and TADBFS have slightly better results than TABFS when the number of evacuees in the building is less than 700. However, TABFS has slightly better results when the number of evacuees in the building is greater than 700.

In Figure 13b, we show the number of time slots needed to evacuate the evacuees. To be more specific, the evacuation speed is compared to these six algorithms. We observe that TABFS can evacuate faster than TASP and TADBFS when the number of evacuees in the building is less than 900. After 900, the number of time slots to evacuate is all 15 for these three algorithms. This indicates that all the emergency exits are on fire after 15 time slots. Based on reports of fire progression rapidly in minutes in [1, 3], we set the duration of one time slot is one minute. Then the golden window for evacuation is 15 min when considering the extreme case of 7 fires. Then, the computational time of the evacuation algorithm is required to be faster than the fire spreading speed when evacuating a group of people. We perform the computational experiments in the computer with a 3.0 GHz AMD Ayzen 7 1700 Eight-core processor with 16 GB RAM. When evacuating 1200 evacuees, the computational time for TABFS and TADBFS are all about ten minutes, and the computational time for TASP is about four hours, which justifies the computational complexity results in Section 5.2. Then only TABFS and TADBFS are the candidate evacuation algorithms. In considering the computational time and group evacuation results in Figure 13, we conclude that TADBFS should be chosen as the evacuation algorithm when the number of evacuees to be evacuated is less than 700. When the number of evacuees to be evacuated is greater than 700, TABFS is recommended to be adopted as the evacuation algorithm.

## 7. Conclusions

A fixed emergency evacuation path is not a valid solution for a fire emergency in the building because of the dynamic fire spreading. The failure probability of evacuation path blocking by the fire is even higher in large buildings because people need more time to evacuate. In this paper, leveraging an IoT sensor to capture the real-time temperature data from the environment, the mathematical model and six algorithms are proposed to identify efficient time-aware evacuation paths in multi-story multi-exit buildings. Three algorithms (BFS, SP, DBFS) do not address the human’s temperature limit constraint, which can be used by the rescue robots or fireman with a fire-proof suit. The other three algorithms (TABFS, TASP, TADBFS) do address the human’s temperature limit constraint, which is to evacuate the evacuees.

In the computational experiments, experiments on evacuating a single evacuee and evacuating a group of evacuees are performed. When evacuating a single evacuee in the open space building and the Taipei 101 building, it is observed that TABFS, TASP and TADBFS identify almost the same evacuation path. Without human temperature limit constraints, BFS, SP and DBFS identify marginal better solutions in terms of total time slots and total temperature on the evacuation path. Another interesting observation is that too low or too high sensor density deployment will incur longer evacuation time and larger temperature on the evacuation path in the Taipei 101 Shopping Mall. TABFS, TASP and TADBFS can help to determine the best sensor density on each floor of the Taipei 101 Shopping Mall.

Computational experiments on evacuating a group of evacuees at the Taipei 101 Shopping Mall are also performed from 100 evacuees to 1200 evacuees. First, the maximum allowable evacuation time is tested in the Taipei 101 Shopping Mall in the extreme case of seven fires. The results show that at most, 15 time slots are allowed to evacuate the people in the building. After 15 time slots, all the eight emergency exits on the first floor will be on fire so that no evacuees can evacuate safely. Considering the case that one time slot is one minute, then the golden window to evacuate the evacuees is 15 min. This is to enforce the computational time of the selected evacuation algorithm should be less than 15 min. Then, only TABFS and TADBFS are qualified because the computation time for these two algorithms is about 10 min to evacuate 1200 people. Next, the experiments on determining the maximum capacity in the Taipei 101 Shopping Mall are also conducted. The results show that there is a capacity limit for the Taipei 101 Shopping Mall where at most 800 evacuees can be successfully evacuated. TABFS can evacuate more people than TADBFS when there are more than 700 people in the building. When the number of people in the building is less than 700, TADBFS should be selected as the evacuation algorithm to evacuate more people than TABFS.

## Data Availability

Data sharing not applicable.
